# Biomaterial-mediated delivery of traditional Chinese medicine ingredients for spinal cord injury: a systematic review

**DOI:** 10.3389/fphar.2024.1461708

**Published:** 2024-10-31

**Authors:** Gang Liu, Zhenzhen Pei, Huizhong Bai, Luyao Huo, Bowen Deng, Shengyuan Jiang, Jingwei Tao, Lin Xu, Jinyu Li, Feng Gao, Xiaohong Mu

**Affiliations:** ^1^ Department of Orthopedics, Dongzhimen Hospital, Beijing University of Chinese Medicine, Beijing, China; ^2^ Guang’an Men Hospital, Chinese Academy of Chinese Medical Sciences, Beijing, China; ^3^ Division of Intelligent and Biomechanical System, State Key Laboratory of Tribology, Department of Mechanical Engineering, Tsinghua University, Beijing, China

**Keywords:** spinal cord injury, traditional Chinese medicine ingredients, biomaterials, hydrogels, scaffolds

## Abstract

**Objective:**

Biomaterials loaded with ingredients derived from traditional Chinese medicine (TCM) are viewed as a promising strategy for treating spinal cord injury (SCI). However, a comprehensive analysis of the existing literature on this topic has not yet been conducted. Therefore, this paper systematically reviews researches related to this approach, aiming to identify gaps and shortcomings in the field.

**Methods:**

PubMed, EMBASE, Web of Science, Chinese Biomedical Literature, Wanfang, and China National Knowledge Infrastructure (CNKI) were searched for retrieving studies on biomaterials loaded with TCM ingredients published from their inception to October 2024. Two reviewers performed screening of search results, information extraction, and literature quality assessment independently.

**Results:**

For this systematic review, 41 publications were included. Six TCM ingredients-paclitaxel, curcumin, tetramethylpyrazine, resveratrol, berberine, and tanshinone IIA were combined with biomaterials for treatment of SCI. Biomaterials were categorized into hydrogels, biodegradable scaffolds, nanoparticles, and microspheres according to the type of scaffold. These drug delivery systems exhibit commendable biocompatibility, drug-loading capacity, and drug-release capabilities, and in combination with TCM ingredients, synergistically contribute to anti-oxidative stress, anti-inflammatory, neuroprotective, and anti-apoptotic effects.

**Conclusion:**

These studies demonstrated the efficacy of biomaterials loaded with TCM ingredients in facilitating motor function recovery and neuroprotection in SCI rats, providing evidence for future research. However, in the complex microenvironment of SCI, achieving the maximum drug loading capacity of TCM ingredients within biomaterials, along with sustained and controlled release to fully exert their pharmacological effects, remains a major challenge for future research.

**Systematic Review Registration:**

https://www.crd.york.ac.uk/PROSPERO/ identifier CRD42024505000.

## Graphical Abstract

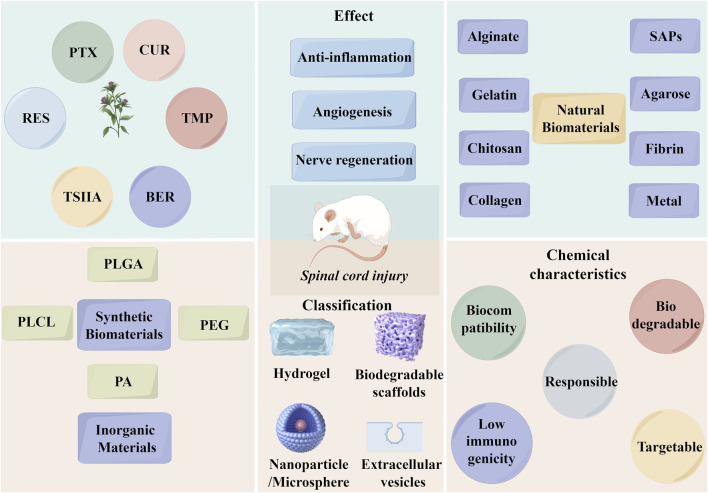

## 1 Introduction

Spinal cord injury (SCI) is a severe central nervous system disorder, with the global incidence of traumatic SCI reaching as high as 900 cases per 1,000,000 people. SCI causes paralysis and severe dysfunction in excretion, posing significant threats to patients’ health and quality of life (Ding et al., 2022). In the early stages of SCI, the primary treatment approach involves surgical intervention combined with high-dose methylprednisolone (MP). Surgery aims to expand the spinal canal to relieve spinal cord compression, while high-dose MP works to reduce secondary oxidative stress and inflammation, both contributing to neuroprotection (Tian et al., 2023). However, the side effects of high-dose MP, such as infections, pneumonia, and femoral head necrosis, cannot be overlooked (Canseco et al., 2021). Moreover, a cohort study found that MP did not provide the expected benefits to SCI patients (Felleiter et al., 2012). Consequently, the routine use of high-dose MP after SCI remains controversial. Therefore, exploring effective alternatives has become a key research direction.

In recent years, research on the use of traditional Chinese medicine (TCM) ingredients for the treatment of SCI has been widely conducted, with increasing focus on their mechanisms in promoting neural repair (Huang et al., 2022; Zhang et al., 2013). The advantages of TCM ingredients in antioxidative stress, anti-inflammatory effects, and reducing neuronal cell death are becoming increasingly evident. However, challenges such as short half-life, low bioavailability, and complex drug intervention targets still need to be addressed. Achieving targeted neural repair and enhancing drug efficacy remain urgent issues to solve (Lu et al., 2020). With advancements in cell biology, materials science, and regenerative medicine, utilizing nanobiomedical technologies to construct scaffolds capable of carrying cells, growth factors, and drugs—such as hydrogels, nanoparticles (NPs), and nanospheres—has emerged as a new approach for treating SCI. Biomaterials possess excellent biocompatibility and support cell adhesion, delivery, and growth. Furthermore, as scaffolds, biomaterials can locally deliver drug payloads to the injury site, enhancing drug utilization (Feng et al., 2023; Pinelli et al., 2022). Therefore, combining biomaterials with TCM ingredients to build drug delivery systems (DDS) offers an effective way to overcome the limitations of single-drug therapies.

Currently, there is a scarcity of systematic evaluations of preclinical research on the use of biomaterials carrying TCM ingredients for SCI treatment. Therefore, to clarify their advantages and limitations, this study, based on previous literature, explored their therapeutic effects from both pharmacological and materials science perspectives, aiming to provide a reference for future research.

## 2 Materials and methods

### 2.1 Registration

This study followed the Preferred Reporting Items for Systematic Reviews and Meta-Analyses (PRISMA) guidelines (Page et al., 2021). Additionally, the research protocol was submitted to PROSPERO for registration (Registration number: CRD42024505000).

### 2.2 Search strategy

Through a literature review, we identified fourteen common TCM ingredients, including curcumin (CUR), resveratrol (RES), tanshinone IIA (TSIIA), tetramethylpyrazine (TMP), baicalin, berberine (BER), rhodioloside, total flavonoids of astragalus, astragaloside, emodin, panax notoginseng saponins, paclitaxel (PTX), and rosmarinic acid. We searched multiple databases, including Chinese Biomedical Literature, Wanfang, China National Knowledge Infrastructure (CNKI), Web of Science, Embase, and PubMed (from inception to October 2024). Relevant papers were identified using search terms such as “curcumin,” “resveratrol,” “tanshinone,” “tetramethylpyrazine,” “baicalin,” “berberine,” “rhodioloside,” “total flavonoids of astragalus,” “astragaloside,” “emodin,” “panax notoginseng saponins,” “paclitaxel,” “rosmarinic acid,” and “spinal cord injury.” We did not restrict the search strategy by biomaterial type due to their diversity. Each paper was individually screened for eligibility. A database-specific approach was applied for each search, without restrictions on blinding methods, languages, or publication dates. Detailed search strategies for each database are provided in Supplementary Table S1.

### 2.3 Eligibility criteria

(1) Types of studies: Animal with SCI were included. Systematic reviews, meta-analyses, case reports, guidelines, clinical studies, and conference proceedings were excluded. (2) Types of participants: The types of animals included encompassed any type of laboratory animals, with no restrictions on age, gender, or strain. (3) Types of intervention: Any type of biomaterials loaded with TCM ingredients was included.

### 2.4 Data collection and quality assessment

Two trained researchers handled the selection of articles and data extraction from studies meeting the inclusion criteria. They performed cross-checks to ensure accuracy. Any discrepancies were resolved by a third researcher. The data extraction process followed a predefined checklist, which covered details such as the author, publication year, country of origin, animal modeling techniques, type of TCM ingredients, biomaterials used, drug delivery methods in animals, and effect.

The quality of the articles included in the analysis was evaluated by two independent reviewers using the SYRCLE Risk of Bias tool for animal research (Hooijmans et al., 2014). The evaluation focused on ten key criteria: 1) sequence generation, 2) baseline characteristics, 3) allocation concealment, 4) random housing, 5) blinded animal intervention, 6) random outcome assessment, 7) blinded outcome assessment, 8) incomplete outcome data, 9) selective outcome reporting, and 10) other types of bias. Any disagreements were addressed with the help of a third reviewer. Each study was classified as having either a “low,” “high,” or “unclear” risk of bias.

### 2.5 Data synthesis

The data from each eligible study were analyzed qualitatively within the main text of the article. A meta-analysis was not conducted due to the observed variability in animal types, models, TCM ingredients, and biomaterials used in the primary studies. As a result, we systematically reviewed and assessed the extracted data, presenting the findings narratively. This approach aimed to evaluate how biomaterials loaded with TCM ingredients support neural repair following SCI.

## 3 Results

### 3.1 Study selection

**FIGURE 1:**
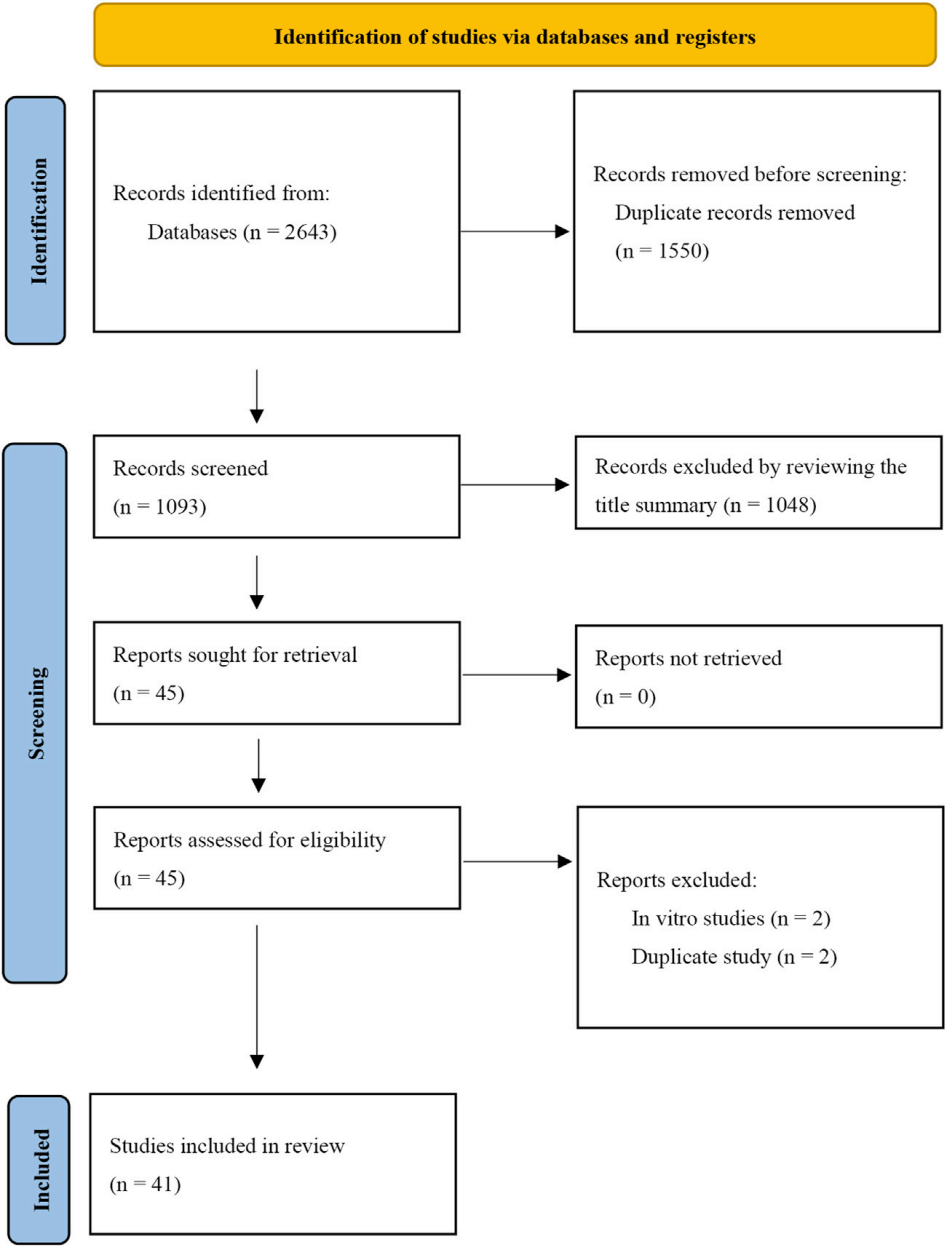
PRISMA flow diagram for identifying eligible studies.

A total of 2,643 records were retrieved from the database search. After removing duplicates, 1,093 records were reviewed. From these, the titles and abstracts of 45 articles were deemed relevant to the objectives of this systematic review. Following a thorough full-text evaluation, 41 articles were selected for qualitative analysis based on the inclusion and exclusion criteria. Four records, initially appearing to meet the inclusion criteria, were excluded after full-text review for the following reasons: two were *in vitro* studies and two were duplicates. The PRISMA flow diagram illustrating the search process used in this systematic review is shown in Figure 1, and the basic information of the included studies is detailed in Table 1.

**TABLE 1:** Basic information of included studies.

Author, year	Country	Animals model	TCM ingredients	Biomaterials	Combination	Pathway	Effect
Xu et al. (2024)	China	SD rats with spinal cord contusion	PTX	TPGS NPs	Idebenone	Intrathecal injection	Inducing neuronal ferroptosis and promoting axon regeneration
Zhu et al. (2024)	China	SD rats with spinal cord contusion	PTX	BSA hydrogel	bFGF	Implantation	Reducing glial scar, and promoting nerve regeneration
Li et al. (2024)	China	SD rats with spinal cord hemisection	PTX	SilMA hydrogel	—	Implantation	Promoting the differentiation of NSCs into neurons
Sun et al. (2023)	China	SD rats with spinal cord transection	PTX	ZIF8 NPs/Collagen hydrogel	—	Implantation	Inducing the differentiation of NSPCs and promoting nerve regeneration
Li et al. (2023)	China	SD rats with spinal cord transection	PTX	PLGA NPs/Collagen scaffold	SDF1α	Implantation	Promoting nerve regeneration and reducing glial scar
Liu et al. (2021a)	China	SD rats with spinal cord transection	PTX	Gelatin hydrogel	SDF1α	Implantation	Promoting nerve regeneration and blood vessels
Yin et al. (2021)	China	Beagle dogs with spinal cord transection	PTX	Collagen scaffold	—	Implantation	Promoting nerve regeneration and reducing glial scar
Liu et al. (2021b)	China	SD rats with spinal cord transection	PTX	Collagen scaffold	NT3	Implantation	Inducing the differentiation of NSCs and promoting nerve regeneration
Nazemi et al. (2020)	Iran	Wistar rats with spinal cord hemisection	PTX	PLGA microspheres/Alginate hydrogel	MH	Implantation	Reducing glial scar and inflammation, and promoting nerve regeneration
Xiao et al. (2020)	China	SD rats with spinal cord contusion	PTX	SAP hydrogel	—	Implantation	Promoting nerve regeneration and reducing glial scar
Liu et al. (2019)	China	Beagle dogs with spinal cord transection	PTX	Collagen scaffold	—	Implantation	Promoting nerve regeneration and reducing glial scar
Liu et al. (2018)	China	SD rats with spinal cord contusion	PTX	Acetalated dextran NPs	—	Implantation	Reducing glial scar and promoting nerve regeneration
Li et al. (2018)	China	SD rats with spinal cord transection	PTX	Collagen scaffold	NSCs	Implantation	Inducing the differentiation of NSPCs and promoting nerve regeneration
Fan et al. (2018)	China	SD rats with spinal cord transection	PTX	Collagen scaffold	Cetuximab	Implantation	Reducing glial scar, promoting nerve regeneration, and inducing the differentiation of NSPCs
Yin et al. (2018)	China	Beagle dogs with spinal cord transection	PTX	Collagen scaffold	—	Implantation	Promoting nerve regeneration and reducing glial scar
Ghaffari et al. (2024)	Iran	Wistar rats with spinal cord hemisection	CUR	BSA NPs/ASCS	—	Implantation	Promoting nerve regeneration and reducing inflammation
Liu et al. (2024)	China	SD rats with spinal cord contusion	CUR	PAH conjugate nanoassemblies	—	Intravenous injection	Promoting nerve regeneration and reducing inflammation
Tang et al. (2024)	China	C57BL/6J mice with spinal cord contusion	CUR	Lipid NPs	—	Intravenous injection	Promoting nerve regeneration and reducing inflammation
Chen et al. (2024)	China	SD rats with spinal cord contusion	CUR	ASCS	NT3	Implantation	Promoting nerve regeneration and reducing inflammation
Ai et al. (2023)	Iran	SD rats with spinal cord contusion	CUR	PLGA NPs/Alginate-gelatin hydrogel	hEnSCs	Implantation	Reducing lesion size
Qian et al. (2023)	China	SD rats with spinal cord contusion	CUR	Polymeric micelles NPs	—	Implantation	Promoting nerve regeneration and reducing inflammation
Su et al. (2023)	China	SD rats with spinal cord contusion	CUR	Metal (Fe^3+^/Cu^2+^)-PPZ NPs	—	Intravenous injection	Reducing inflammation and neuronal apoptosis
Zhang (2022)	China	SD rats with spinal cord transection	CUR	Chitosan NPs/Sodium alginate hydrogel	—	Implantation	Inducing the differentiation of EMSCs into neurons and neural stem cells, reducing glial scar and promoting neural regeneration
Bonilla et al. (2021)	Spain	SD rats with spinal cord contusion	CUR	PA NPs	—	Intrathecal injection	Reducing inflammation and promoting nerve regeneration
Li et al. (2021)	China	SD rats with spinal cord transection	CUR	PLCL microspheres/Chitosan- hydrogel	—	Implantation	Reducing inflammation
Krupa et al. (2019)	Czech Republic	Wistar rats with compression	CUR	Lipid NPs	—	Implantation	Promoting nerve regeneration and reducing glial scar
Requejo-Augular et al. (2017)	Spain	SD rats with spinal cord contusion	CUR	PA NPs	—	Intrathecal injection	Reducing glial scar, inflammation, and cell death
Wu et al. (2014)	China	Kunming mice with spinal cord contusion	CUR	PEG-DSPE micelles NPs	—	Intravenous injection	Enhancing neural targeting
Wu et al. (2024)	China	SD rats with spinal cord contusion	TMP	Agarose hydrogel	—	Implantation	Reducing inflammation
Jiang et al. (2023)	China	SD rats with spinal cord transection	TMP	Gelatin hydrogel	—	Implantation	Reducing inflammation and promoting axon regeneration
Deng et al. (2024)	China	SD rats with spinal cord transection	TMP	Gelatin hydrogel	—	Implantation	Enhancing the regeneration of blood vessels
Lin et al. (2021)	China	SD rats with spinal cord contusion	TMP	Human serum albumin NPs	—	Intravenous injection	Enhancing neural targeting
Li et al. (2021)	China	SD rats with spinal cord contusion	TMP	Human serum albumin NPs	—	Intravenous injection	Enhancing neural targeting, reducing oxidative stress and inflammation
Li et al. (2022)	China	C57BL/6 J mice with spinal cord contusion	RES	Hollow manganese dioxide NPs	—	Intravenous injection	Reducing oxidative stress, inflammation and neuronal apoptosis
Jiang et al. (2021)	China	C57BL/6 J mice with spinal cord contusion	RES	Manganese-doped silica NPs	—	Intravenous injection	Reducing oxidative stress, apoptosis, and inflammation
Chen et al. (2020)	China	Wister rats with spinal cord ischemia-reperfusion	RES-PUE	PLGA NPs	—	Intravenous injection	Reducing oxidative stress
Wang et al. (2023)	China	SD rats with spinal cord hemisection	BER	Small extracellular vesicles/Gelatin hydrogel	—	Implantation	Promoting nerve regeneration and reducing glial scar
Mahya et al. (2021)	Iran	Wistar rats with spinal cord compression	BER	Chitosan NPs/Alginate-chitosan hydrogel	—	Implantation	Reducing lesion size
Li et al. (2022)	China	SD rats with spinal cord contusion	TSIIA	PLGA microspheres	—	Intrathecal injection	Reducing inflammation
He (2016)	China	SD rats with spinal cord transection	TSIIA	Fibrin hydrogel	—	Implantation	Promoting orderly axon regeneration and blood vessels
Rao et al. (2022)	China	SD rats with spinal cord contusion	APS-TSIIA	Selenium NPs		Intraperitoneal injection	Reducing oxidative stress

Note: ASCS, acellular spinal cord scaffold; APS, astragalus polysaccharidesis; BSA, bovine serum albumin; bFGF, basic fibroblast growth factor; BER, berberine; CUR, curcumin; EMSCs, Ectodermal mesenchymal stem cells; hENSCs, Human endometrial stem cells; MH, minocycline hydrochloride; NSPCs, Neural stem/progenitor cells; NSCs, Neural stem cells; NT3, Neurotrrophic factor 3; NPs, Nanoparticles; PAH, polypeptide with hydrazide groups; PTX, paclitaxel; PUE, puerarin; PLGA, Poly (lactic-co-glycolic acid); PLCL, Poly (L-lactic acid)-polycaprolactone; PEG-DSPE, polyethyleneglycolcarbamyl distearoylphosphatidyl ethanolamine micelles; PPZ, polyphosphazene; PA, Polyacetals. RES, resveratrol; SDF1α, Stromal cell-derived factor-1α; SAP, self assembled peptide; TPGS, d-α-tocopheryl polyethylene glycol 1,000 succinate; TMP, tetramethylpyrazine; TSIIA, Tanshinone IIA; ZIF-8, Zeolitic imidazolate framework-8.

### 3.2 Study quality assessment

**FIGURE 2:**
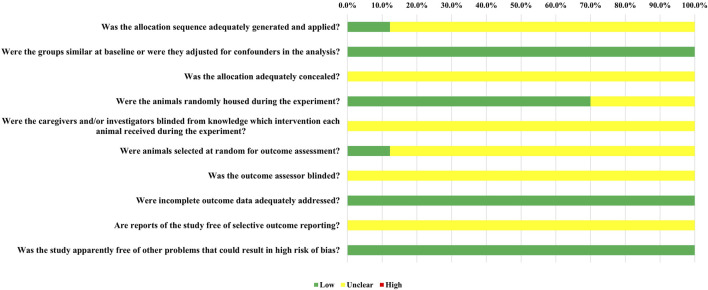
The results of the risk of bias assessment.

We utilized SYRCLE’s Risk of Bias (RoB) tool to evaluate the risk of bias in each individual study. The results of this assessment are illustrated in Figure 2. All studies were generally assessed as having a low risk of bias; however, some or all of the studies did not report on certain domains. The RoB tool provided a total of 410 entries across the ten relevant signaling questions. Among these, 158 entries indicated a low risk of bias, 252 entries showed an unclear risk of bias, and none revealed a high risk of bias.

Overall, 5 out of the 41 randomized controlled trials (12.2%) provided evidence that randomization was performed using a random number table or computer. Nonetheless, these studies did not report the use of concealed allocation. All studies reported that the baseline characteristics, including animal species and sex, were appropriately matched. In terms of allocation concealment (Item 3), all studies were classified as having an unclear risk of bias due to the lack of reporting. Twenty-five studies (70.0%) were assessed as having a low risk of bias for random housing. Due to insufficient information, all studies were deemed to have an unclear risk of bias regarding investigator blinding, blinding of outcome assessors, and selective outcome reporting. For random outcome assessment (Item 6), 5 studies (12.2%) had a low risk of bias. Additionally, all studies were found to have a low risk of bias concerning incomplete outcome data.

### 3.3 Characteristics of included studies

A total of 41 studies were included in the review, with 34 studies originating from China, 4 from Iran, 2 from Spain, and 1 from the Czech Republic. The studies investigated 6 different TCM ingredients: PTX, CUR, TMP, RES, BER, and TSIIA. Specifically, 15 studies focused on PTX, 13 on CUR, 5 on TMP, 2 on RES, 2 on BER, and 3 on TSIIA.

The biomaterials used to deliver TCM ingredients comprised both natural materials (such as collagen, gelatin, alginate, chitosan, agarose, polypeptide, and fibrin) and synthetic materials [including poly (lactic-co-glycolic acid) (PLGA), polyacetals (PA), polyphosphazen (PPZ), polyethyleneglycolcarbamyl distearoylphosphatidyl ethanolamine (PEG-DSPE), and poly (L-lactic acid)-polycaprolactone (PLCL)]. Additionally, nanoparticles (NPs) and microspheres made from inorganic materials were utilized. These biomaterials were categorized into hydrogels, biodegradable scaffolds, NPs, and microspheres based on their types.

Regarding animal models, except for 2 studies that used beagles, all other studies employed rats. The SCI models included transection, hemisection, contusion, compression, and ischemia-reperfusion. Administration methods varied and included implantation, injury-site injection, intrathecal injection, intraperitoneal injection, and intravenous injection. Transection and hemisection models were predominantly used for research involving implantable materials, such as hydrogels and biodegradable scaffolds, which were implanted into the exposed area following spinal cord transection or hemisection. The other models were primarily used for studying injectable materials. Research primarily addressed aspects such as inflammation, oxidative stress, glial scar formation, neural stem cell differentiation, axon regeneration, and neuroprotection.

## 4 Discussion

### 4.1 Pathophysiology

SCI is a complex condition and can be classified into primary and secondary SCI based on disease progression (Venkatesh et al., 2019). Primary SCI refers to the initial mechanical trauma to the spinal cord, including acute stretching, traction, compression, and axonal shearing. In contrast, secondary SCI is a progressive process that follows primary SCI and involves a cascade of pathological reactions, such as inflammation, oxidative stress, vascular damage, and cell death (Flack et al., 2022). Secondary SCI often results in more extensive damage than primary SCI and can be divided into acute, subacute, and chronic phases.

Inflammation plays a crucial role during the acute and subacute phases, involving multiple cell types such as neutrophils, microglia, macrophages, B/T lymphocytes, and various inflammatory and chemotactic factors. After SCI, resident microglia become activated, followed by the recruitment of neutrophils, macrophages, and other immune cells to the injury site. Neutrophils peak within the first 24 h post-injury (Filipp et al., 2019; Popovich et al., 1997; Cao et al., 2022). Initially, macrophages are derived from microglia, but they are gradually replaced by monocyte-derived macrophages recruited to the injury site. Macrophages infiltrate the site approximately 48 h post-SCI and reach their peak between 7–10 days (Thawer et al., 2013; London et al., 2013). During this process, microglia and macrophages can adopt either a pro-inflammatory (M1) or anti-inflammatory (M2) phenotype. The M1-like response persists into the subacute and chronic phases, while the M2-like response is transiently present during the subacute phase (Devanney et al., 2020). Promoting the M2-like phenotype may be essential for suppressing inflammation following SCI. Vascular regeneration begins early in secondary SCI and involves the proliferation and migration of pericytes and endothelial cells (Zamanian et al., 2023). Glial scar formation is a defining feature of the chronic phase and is primarily related to astrocyte activation. The glial scar acts as a physical and chemical barrier, impeding axonal regeneration at the injury site (Clifford et al., 2023).

In summary, inflammation, vascular regeneration, neural regeneration, and glial scar formation are key biological processes involved in SCI. Encouragingly, tissue-engineering materials incorporating TCM ingredients have shown promise in addressing these aspects.

### 4.2 Paclitaxel

PTX is a major anticancer drug found in the bark of *Taxus spp*, which can inhibit mitosis and stabilize microtubule formation. It also plays a role in promoting neuronal differentiation and axon regeneration. PTX is poorly soluble in water and most commonly used pharmacological solvents, and it has difficulty crossing the blood-spinal cord barrier (BSCB), which limits its application in SCI (Huang et al., 2022). Designing DDS of PTX is an effective approach to address this issue.

Due to the complex pathological mechanisms of SCI, combining PTX with other molecules in DDS to work synergistically become an effective approach. Researchers co-loaded PTX liposomes and stromal cell-derived factor-1α (SDF1α) into a collagen-based photosensitive hydrogel (Liu et al., 2021a). SDF1α promoted wound healing by recruiting mesenchymal stem cells and endothelial progenitor cells to enhance neovascularization, but it had been rarely reported in the field of SCI (Zhao W. et al., 2017). Research results indicated that PTX-SDF1α/photosensitive hydrogel could promote axon regeneration and exert neuroprotective effects. SDF1α also facilitated the migration of human umbilical vein endothelial cells, enhancing angiogenesis. Long-term observation revealed that their synergistic effects improved the local microenvironment of the injury, reduced glial scar formation, and promoted the improvement of rat motor function. Li et al. (2023) employed a similar design concept by constructing a PTX@PLGA-SDF1α/collagen porous scaffold. They replaced the liposomes loaded with PTX in the previous study with PLGA NPs. PTX modified with PLGA NPs increased solubility, stability, and prolonged drug release time. The study showed that SDF1α recruited neural stem cells and promoted their aggregation in the injury site, while PTX induced neural stem cells to differentiate into neurons rather than astrocytes, ultimately inhibiting glial scar formation. Nazemi et al. (2020) also constructed PTX@PLGA microspheres, which they loaded into agarose hydrogel along with minocycline hydrochloride. Minocycline hydrochloride improved the local microenvironment by inhibiting inflammation, and together with PTX, it promoted nerve regeneration. Idebenone, as an antioxidant, exerted neuroprotective effects by inhibiting neuronal ferroptosis. Combining idebenone with PTX is also a novel approach for synergistic drug delivery. Xu et al. (2024) conjugated idebenone with the PTX using an acid-sensitive, self-immolative ketal linker to create a heterodimeric prodrug. This prodrug was then formulated into a nanomedicine with a high drug load by incorporating chondroitin sulfate proteoglycan-binding peptide-modified (tetrapeptide cysteine-alanine-lysine-glutamine, or CAQK) d-α-tocopheryl polyethylene glycol 1,000 succinate (TPGS). In this work, TPGS was used as a nanomedicine carrier to load PTX and idebenone. Additionally, the researchers modified the nanocarrier with CSPG-targeting peptides to further enhance drug efficacy. Zhu et al. (2024) co-loaded PTX and basic fibroblast growth factor into a hydrogel prepared from bovine serum albumin. The combination of PTX and basic fibroblast growth factor can promote axonal growth through the glial scar and inhibit the formation of reactive astrocytes. Therefore, the design mode of PTX combined with other factors or cells may be an effective means of synergy and enhancement. Additionally, Li et al. (2024) designed a 3D-printed porous SilMA hydrogel scaffold loaded with PTX. Xiao et al. (2020) used a biologically functionalized self-assembled peptide nanofiber scaffold (FGLmx/Taxol), while Liu et al. (2018) utilized dextran-based biodegradable nanoparticles, providing new approaches for PTX treatment of SCI.

Researchers from the Chinese Academy of Sciences, led by Li et al. (2018), conducted a series of studies on PTX DDS. They developed a functional collagen microchannel scaffold by encapsulating PTX liposomes and neural stem cells. *In vitro* experiments revealed the highest neuronal differentiation rate at 4.25 ng/mL PTX, while *in vivo* results showed the most significant neuronal regeneration with 256 ng PTX. This study highlighted PTX’s effect on neural stem cell differentiation and its link to the Wnt/β-catenin pathway. To explore better drug carriers, the researchers utilized bovine aponeurosis to prepare a linear ordered collagen scaffold (LOCS), also known as the NeuroRegen scaffold, which guides axonal growth. Preclinical and clinical studies confirmed its effectiveness (Xiao et al., 2018; Zhao Y. et al., 2017; Han et al., 2014). PTX-loaded LOCS (PTX/LOCS) further improved motor function in animal models, with enhanced neuronal regeneration and synaptic formation at the injury site. However, no increase in the cortical spinal tract was observed, suggesting that recovery relied on newly formed neuronal circuits rather than long-tract regeneration (Liu et al., 2019; Yin et al., 2018; Steward et al., 2008). An innovative study demonstrated that scar tissue removal followed by PTX/LOCS implantation 3 months post-injury significantly promoted axon regeneration, neurogenesis, and functional recovery. This outcome was attributed to scar removal activating endogenous neural stem cells and creating a favorable microenvironment for differentiation (Yin et al., 2021). Additionally, combining PTX/LOCS with neurotrophic factor 3 and cetuximab produced similar benefits (Liu et al., 2021b; Fan et al., 2018). In their latest work, Sun et al. (2023) developed a cascade-responsive DDS using modularly designed “egg” NPs. PTX was encapsulated in a zeolitic imidazolate framework-8 (ZIF-8) to form ZP-PTX. The surface was coated with tannic acid, Fe³⁺, and tetradecanol to create ZPT-PTX, which was incorporated into a collagen hydrogel (Col-ZPT-PTX). Near-infrared light triggered heat generation, releasing PTX from ZIF-8, while the acidic SCI environment facilitated further drug release. This dual-responsive system promoted neurogenesis and improved motor function recovery.

In summary, PTX primarily promotes SCI repair by enhancing neural stem cells differentiation into neurons, synaptic formation, and axon regeneration, while inhibiting glial scar formation. One of its most important mechanisms is the promotion of mature neuronal differentiation. The various forms of DDS can be categorized as NPs, microspheres, hydrogels, bio-scaffolds, and combinations of NPs or microspheres with scaffolds or hydrogels. In conclusion, the advances in DDS design for PTX has achieved a series of progress. The successful development of these materials and the exploration of PTX’s underlying mechanisms for SCI repair offer valuable insights for future research.

### 4.3 Curcumin

CUR is a hydrophobic polyphenolic compound primarily found in the rhizomes of turmeric. A major limitation of CUR is its low bioavailability, with minimal concentrations detected in the serum and tissues, largely due to extensive metabolism in the liver and intestines, as well as rapid excretion (D’Souza and Devarajan, 2016). To address these challenges, NPs and microspheres have been employed in the DDS of CUR. Su et al. (2023) developed a metal (Fe/Cu)-CUR-polyphosphates NPs, significantly enhancing the loading efficiency and water solubility of CUR. This system exhibited synergistic anti-inflammatory and neuroregenerative effects for SCI treatment, with research indicating that CUR’s early anti-inflammatory and anti-apoptotic actions were mediated via the Wnt/β-catenin pathway. Krupa et al. (2019) reported highly pure CUR into Lipodisq™, a lipid-based discoidal NP. These biodegradable NPs are suitable for both local and systemic applications, demonstrating efficacy in inhibiting glial scar formation, promoting axon regeneration, and reducing inflammation.

However, localized drug release remains a challenge due to cerebrospinal fluid circulation, limiting the therapeutic efficacy of NPs alone. To overcome this, researchers combined CUR-NPs with hydrogels. Ai et al. (2023) prepared CUR-PLGA NPs, which were then loaded into alginate/gelatin hydrogels. Other researchers developed nanospheres containing CUR using PLCL and loaded them into chitosan/β-glycerophosphate hydrogels (Li, 2021). Ghaffari et al. (2024) prepared CUR-loaded bovine serum albumin NPs and incorporated them into an acellular spinal cord scaffold. This natural biomaterial, derived from animal spinal cords, retained the extracellular matrix and original tissue structure after all cellular components were removed. Chen et al. (2024) also employed an acellular spinal cord scaffold and co-loaded CUR with neurotrophin-3 to enhance therapeutic outcomes. The studies reviewed demonstrate that using DDS strategies combining NPs and hydrogels enhances localized CUR release, significantly amplifying its anti-inflammatory effects.

After SCI, the microenvironment becomes highly complex, making controlled drug release tailored to these conditions a critical challenge. Bonilla et al. (2021) developed a pH-responsive polyacetal-CUR nanoconjugate, enabling continuous CUR release, with rapid release achieved under low pH conditions. Their research indicated that CUR could increase M2-type microglial cell expression and inhibit neuroinflammation, potentially through the NF-κB signaling pathway. Requejo-Aguilar et al. (2017) employed a pH-responsive polymer-CUR conjugate, demonstrating the highest release rate at pH 5.5. Their findings also revealed that CUR inhibited the Rho/Rock pathway, promoting axon regeneration, a novel discovery in SCI treatment. In another study, researchers conjugated PEGylated polypeptides with hydrazide groups to CUR using 4-formylphenylboronic acid as a linker. The resulting conjugate underwent *in situ* self-assembly to form nanoassemblies responsive to low pH and elevated reactive nitrogen and oxygen species (Liu et al., 2024). All three studies employed polymer conjugation to develop DDS, providing new insights for SCI therapy. Chemical bonds played a crucial role, forming stable structures that facilitated responsive drug release in reaction to various physical or chemical environments, enabling controlled drug delivery.

Beyond pH sensitivity, the exploration of temperature-responsive materials has also gained attention. Qian et al. (2023) created dual-sensitive micelle NPs with a hydrophilic exterior and CUR-encapsulated interior through block copolymer assembly. This design enhanced hydrophilicity and enabled improved release in response to changes in temperature and pH, significantly boosting bioavailability. Drug release tests revealed that in an acidic environment (pH 5.5) and elevated temperature (37°C), CUR achieved the highest release rate, aligning with the post-SCI microenvironment. Animal experiments further confirmed that CUR modulated microglial/macrophage polarization, increasing the proportion of M2-type cells and suppressing inflammation. Additionally, researchers observed reductions in cavity size and glial scar formation at the injury site. These effects were attributed to CUR’s anti-inflammatory properties, creating a favorable environment for neural regeneration. CUR also facilitated the growth of neurons and oligodendrocytes while reducing the production of astrocytes, further promoting recovery.

Targeted DDS have also been applied in the treatment of SCI using CUR. Researchers designed a drug-carrying polymeric micelle with neuro-targeting capabilities, using PEG-DSPE as the drug-carrying materials, and CUR as the therapeutic drug. Apamin, a primary component of bee venom known for its neural targeting effects (Zhang et al., 2015), was conjugated with N-hydroxysuccinimide-PEG-DSPE in a site-specific manner (Wu et al., 2014). Compared to polymeric micelles without apamin, the drug distribution in the spinal cords of SCI rats was significantly improved following intervention with Apamin-CUR-PEG-DSPE, suggesting this as an effective measure to enhance drug targeting to the nervous system. In another work, CUR-loaded liposomes were used as the core and coated with platelet-neutrophil hybrid vesicles, forming deoxyribonuclease I-modified hybrid membrane-coated nanoparticles. These nanoparticles regulate the neuroinflammatory microenvironment by targeting neutrophils, thereby eliminating neutrophil extracellular traps and reducing proinflammatory cytokines, aiming to achieve effective treatment of SCI (Tang et al., 2024). Developing targeted DDS can effectively enhance drug utilization and represents a promising design strategy.

### 4.4 Tetramethylpyrazine

Tetramethylpyrazine (TMP), a monomeric alkaloid with the molecular formula C₈H₁₂N₂, is derived from the dried rhizomes of *Ligusticum wallichii*, a member of the umbelliferae family. TMP is widely used in treating cardiovascular and cerebrovascular diseases (Lin et al., 2022). In our study, we included five research projects focusing on DDS of TMP for SCI treatment. Researchers developed conductive hydrogels by polymerizing agar with polypyrrole and loading it with TMP (Wu et al., 2024). Researchers constructed a TMP-polypyrrole-gelatin methacryloyl hydrogel using a similar approach (Jiang et al., 2023; Deng et al., 2024). The results from these studies demonstrated that conductive hydrogels can enhance bi-electrical signal communication between cells and restore the disrupted conductive neural pathways caused by injury, while maintaining the electrophysiological microenvironment required for neural regeneration. DDS of TMP inhibited inflammation, promoted axonal regeneration, and exerted neuroprotective effects.

Targeted DDS have also been developed for TMP. Reserachers conjugated the HIV trans-activator of transcription (TAT) peptide to human serum albumin NPs, creating TAT-TMP-NPs capable of being internalized by neutrophils and delivered to SCI lesions. Neutrophils, as an essential component of the innate immune system, are among the first responders at the injury site following SCI. The TAT peptide, known for targeting neutrophils, enhances their uptake of TMP-NPs. These NPs are then transported to the injury site along with the migrating neutrophils, helping overcome the BSCB. *In vivo* imaging studies demonstrated that the distribution of TAT-TMP-NPs within the spinal cord was significantly greater than that of the control group. The study further confirmed TMP’s role in reducing inflammation and mitigating oxidative stress, providing additional therapeutic value for SCI treatment (Lin et al., 2021; Li et al., 2021).

### 4.5 Resveratrol

Resveratrol (RES), a natural polyphenol, is found abundantly in grapes, pine, *polygonum cuspidatum*, *cassia* seeds, peanuts (He et al., 2022). It is widely recognized for its antioxidant, anti-inflammatory, and neuroprotective properties. However, despite its potential in treating central nervous system diseases, RES faces a significant challenge: limited ability to cross the BSCB, restricting its therapeutic application. To address this limitation, Li et al. (2022) designed two types of DDS to enhance RES delivery to the injury site. In one study, they utilized chitosan-modified hollow manganese dioxide NPs to transport RES. While hollow manganese offers a large cavity suitable for carrying insoluble drugs, it alone is insufficient to penetrate the BSCB. Chitosan, with its abundant positive charges, was applied to modify the surface of the NPs, facilitating interaction with the negatively charged membranes of capillary endothelial cells in the central nervous system, thus aiding BSCB penetration (Cortés et al., 2020). Another study took advantage of silica’s BSCB-crossing ability and designed plasma complex component-functionalized manganese-doped silica NPs with redox-responsive properties (Jiang et al., 2021). Both studies demonstrated that RES effectively inhibits oxidative stress, inflammation, and apoptosis following SCI. In addition, Chen et al. (2020) developed PLGA NPs encapsulating both RES and puerarin. Their findings revealed that the combined application of these two herbal ingredients significantly reduced oxidative stress post-SCI, further enhancing their neuroprotective potential.

### 4.6 Berberine

Berberine (BER) is the primary bioactive ingredient in *Rhizoma coptidis* (Feng et al., 2019). Despite its significant ability to regulate the local microenvironment, BER’s limited solubility and permeability across the BSCB greatly restrict its systemic effectiveness (Habtemariam, 2020). Wang et al. (2023) addressed these challenges by loading BER onto small extracellular vesicles (sEVs) derived from human umbilical cord mesenchymal stem cells, creating sEVs-BER NPs. These vesicles could significantly enhance the solubility of BER. To achieve localized release, the sEVs-BER NPs were incorporated into a gelatin methacryloyl hydrogel at the injury site. The study found that BER inhibited local inflammation and reduced fibrosis in the SCI microenvironment. In another approach, Mahya et al. (2021) encapsulated BER within chitosan NPs, which were then integrated into a hybrid alginate-chitosan hydrogel. The previously mentioned ability of chitosan to penetrate the BSCB was utilized in this design. Both studies demonstrated that combining NPs with hydrogels for BER delivery resulted in positive therapeutic outcomes, highlighting the potential of these DDS strategies for SCI treatment.

### 4.7 Tanshinone IIA

Tanshinone IIA (TSIIA), the primary active component of *Danshen*, has demonstrated potential therapeutic effects on neurological diseases linked to oxidative stress, such as SCI (Yang et al., 2017). Li (2022) encapsulated TSIIA in PLGA slow-release microspheres and investigated its effects on SCI in rats through intrathecal injection. Their research revealed that TSIIA alleviated inflammation by promoting the expression of M2 microglia through the inhibition of the Notch signaling pathway. He (2016) developed a fibrin hydrogel loaded with TSIIA, featuring a multi-level directional structure that provided a framework for guided axonal growth. The results indicated that TSIIA not only facilitated orderly axonal growth but also repaired microvascular damage. In another study, researchers applied inorganic nanoparticle technology to develop selenium nanoparticles carrying both TSIIA and Astragalus polysaccharides. Selenium nanoparticles are promising carriers for antioxidant or anti-inflammatory drugs due to their low toxicity, good biocompatibility, and antioxidative properties. The study showed that these selenium nanoparticles combined with the antioxidative effects of TSIIA and Astragalus polysaccharides, significantly inhibited oxidative stress following SCI (Rao et al., 2022).

### 4.8 Summary of biomaterials types

TCM ingredients hold significant potential in treating SCI, but their poor solubility and limited ability to cross the BSCB present major challenges for effective therapy. To address these limitations, various biomaterials have been employed to construct DDS for TCM ingredients, categorized into hydrogels, biodegradable scaffolds, NPs, and microspheres. Hydrogels, formed by cross-linked hydrophilic polymers, retain large amounts of water and can be designed to mimic the mechanical properties of human tissues, making them ideal for drug delivery. Encapsulating drugs within the matrix helps prevent rapid proteolytic degradation, extend the release period, and enable localized delivery, making hydrogels particularly effective for SCI treatment (Wang et al., 2022; Shan and Wu, 2023).

Hydrogel-based DDS can be divided into two types: one uses chemical bonds to cross-link TCM ingredients directly within the hydrogel matrix, while the other involves encapsulating TCM ingredients in NPs or microspheres, which are then embedded into the hydrogel. The combination of hydrogels with NPs or microspheres supports staged drug release, extending the release period and offering insights for layered or multi-stage drug delivery designs.

The literature identifies conductive hydrogels and injectable hydrogels as two important categories. Conductive hydrogels assist in transmitting electrical signals and maintaining the endogenous electrical microenvironment crucial for neural repair, while injectable hydrogels allow *in-situ* application at the injury site, minimizing further damage by adapting to irregular wounds. Additionally, polymer scaffolds like fibrin and collagen have been explored as carriers for TCM ingredients. These scaffolds’ porous structures, with varying pore size and porosity, significantly impact axonal regeneration, demonstrating the potential of these biomaterials in SCI therapy. Integrating TCM with advanced DDS offers promising strategies for improving drug stability, targeted delivery, and overall therapeutic outcomes for SCI.

### 4.9 Composite drug delivery systems

SCI presents a highly complex microenvironment that evolves both spatially and temporally, involving dynamic processes such as inflammation, vascular regeneration, and neural regeneration. In such a complex setting, the therapeutic efficacy of a single plant extract or molecule is often limited. As a result, composite DDS have emerged as a promising strategy, combining TCM ingredients with other molecules, drugs, or cells to create synergistic therapeutic effects. For example, in the development of DDS for PTX, the integration of neural stem cells enhances the ability of PTX to promote neural stem cells differentiation into neurons. This approach leverages synergistic interactions between components, making the therapeutic strategy more effective in addressing the multiple challenges posed by SCI. This trend reflects the multidisciplinary and innovative nature of current research, emphasizing the importance of composite systems that address the diverse pathological mechanisms of SCI. Combining ingredients and therapeutic agents in DDS aligns with the goal of layered and responsive delivery, ensuring more targeted and sustained treatment in the complex microenvironment of SCI.

### 4.10 Responsive drug delivery systems

Regenerative medicine requires precise control over drug release, as traditional DDS, which primarily focus on extending drug half-life and slow release, often fail to maximize therapeutic outcomes. The development of responsive materials that adapt to changes in the post-SCI microenvironment or allow the sequential release of multiple drugs has become essential.

Recent research emphasizes stimuli-responsive DDS that can react to environmental triggers such as pH, temperature, redox gradients, light, magnetic fields, and ultrasound, dynamically altering their chemical or physical properties to enhance drug release (Qu et al., 2020). This concept has also been applied to DDS carrying TCM ingredients, including pH-, temperature-, radical-, and light-responsive systems. For example, Sun et al. (2023) developed a combined DDS using NPs and hydrogels loaded with PTX, employing both light and pH responsiveness to achieve staged drug release. Such responsive designs hold great potential for the future construction of multi-ingredient DDS.

Different phases of SCI demand tailored interventions. In the acute phase, the focus is on anti-inflammation, while in the chronic phase, the priority shifts to promoting neural regeneration and inhibiting glial scarring. A DDS capable of sequentially releasing multiple TCM ingredients at appropriate stages would harness their full therapeutic potential, providing a more targeted and effective treatment strategy for SCI.

### 4.11 3D printing

3D printing has gained increasing attention in tissue engineering due to its ability to rapidly fabricate complex delivery platforms. Koffler et al. (2019) utilized a microscale continuous projection printing method, capable of producing 3D biomimetic hydrogel scaffolds tailored to fit a rat’s spinal cord in just 1.6 s, offering a precise approach to enhance central nervous system regeneration through precision medicine. Similarly, Zhang (2022) applied this technology to develop DDS for TCM ingredients, yielding promising results.

The use of 3D printing to create scaffolds with intricate and precise structures marks a key direction for future advancements. This technology’s ability to fabricate patient-specific, customized structures paves the way for targeted and efficient treatments in SCI and other medical conditions, expanding the potential for personalized medicine.

### 4.12 Blood-spinal cord barrier

The BSCB presents a significant obstacle in SCI treatment, limiting drug permeability and reducing therapeutic efficacy (Tran et al., 2018). Enhancing drug delivery across the BSCB has therefore become a critical focus in research. In studies on DDS for TCM ingredients, researchers have incorporated targeting molecules to improve drug distribution at the injury site. However, addressing the BSCB barrier is primarily relevant to intravenous administration in preclinical studies.

A review of included studies highlights that implantable materials for SCI repair often use spinal cord transection or hemisection models, where the BSCB is already compromised before drug delivery. Similarly, in studies utilizing injectable materials, researchers often adopt spinal cord contusion models, with intrathecal administration delivering drugs directly below the dura mater at the injury site. This method bypasses the BSCB, ensuring the drug reaches the injury site via cerebrospinal fluid circulation. Intrathecal injection—where drugs are administered directly into the cerebrospinal fluid—provides a targeted approach to overcome BSCB limitations.

Given that most TCM ingredients face challenges crossing the BSCB, future research should focus on enhancing DDS targeting or optimizing administration methods through appropriate experimental models and delivery routes.

## 5 Conclusion

This study comprehensively incorporates and summarizes research related to the DDS of TCM ingredients, thoroughly exploring the potential pharmacological mechanisms of six TCM ingredients: PTX, CUR, TMP, RES, BER, and TSIIA in the treatment of SCI. It also analyzes the advantages and limitations of DDS. Overall, significant progress has been made in developing DDS of TCM ingredients, paving the way for further research. However, several challenges remain. Firstly, the optimal drug loading and release rates are still uncertain, particularly the ideal concentration of TCM ingredients required at different stages of SCI. Secondly, the optimal phase for slow-release remains unclear, and relying solely on *in vitro* experiments to determine release rates is insufficient. Thirdly, many studies emphasize the physicochemical properties of biomaterials and track neural regeneration indicators, treating TCM ingredients primarily as anti-inflammatory or antioxidant agents. This overlooks their more complex therapeutic mechanisms in SCI treatment, limiting the scope of their potential applications. In addition, when constructing DDS with new materials, biotoxicity should be given more thorough consideration, and more comprehensive testing methods should be implemented, such as hemocompatibility testing, degradation and metabolic pathway analysis, and immune response testing. This goes beyond merely conducting *in vitro* compatibility tests and single-organ morphological observations in animals. Lastly, concluding that neural regeneration has been achieved based solely on the increase in neurons or morphological improvements in the injury site is not sufficiently rigorous. Deeper exploration of the underlying mechanisms is essential to ensure accurate findings. With advancements in pharmacology and materials science, these challenges are expected to be systematically addressed, paving the way for better exertion of the pharmacological effects of TCM ingredients and improving SCI treatment effects.

## Data Availability

The original contributions presented in the study are included in the article/Supplementary Material, further inquiries can be directed to the corresponding authors.
