# Supplementary material for: Biomaterial-mediated delivery of traditional Chinese medicine ingredients for spinal cord injury: a systematic review
Source: Front Pharmacol. 2024 Oct 31;15:1461708. doi: 10.3389/fphar.2024.1461708 (PMC11560789; doi:10.3389/fphar.2024.1461708)
Supplement: Supplementary file 1 [file Table1.DOCX]

**Table S1: Chinese and English search strategies**

| **PubMed**  (("Spinal Cord Injuries"[MeSH Terms] OR ("Spinal cord injury"[Title/Abstract] OR "Spinal injury"[Title/Abstract] OR "Spinal Cord Trauma"[Title/Abstract] OR "cord trauma spinal"[Title/Abstract] OR "Spinal Cord Traumas"[Title/Abstract] OR "trauma spinal cord"[Title/Abstract] OR "myelopathy traumatic"[Title/Abstract] OR "Traumatic Myelopathies"[Title/Abstract] OR "Traumatic Myelopathy"[Title/Abstract] OR "injuries spinal cord"[Title/Abstract] OR "cord injuries spinal"[Title/Abstract] OR "cord injury spinal"[Title/Abstract] OR "injury spinal cord"[Title/Abstract] OR "Spinal Cord Transection"[Title/Abstract] OR "Spinal Cord Laceration"[Title/Abstract] OR "laceration spinal cord"[Title/Abstract] OR "post traumatic myelopathy"[Title/Abstract] OR "post traumatic myelopathy"[Title/Abstract] OR "Post-Traumatic Myelopathies"[Title/Abstract] OR "contusion spinal cord"[Title/Abstract] OR "contusions spinal cord"[Title/Abstract] OR "cord contusions spinal"[Title/Abstract] OR "Spinal Cord Contusions"[Title/Abstract] OR "Spinal Cord Contusion"[Title/Abstract])) AND (("Curcumin"[MeSH Terms] OR ("turmeric yellow"[Title/Abstract] OR "yellow turmeric"[Title/Abstract] OR "curcumin phytosome"[Title/Abstract] OR "Diferuloylmethane"[Title/Abstract]) OR ("Ginsenosides"[MeSH Terms] OR ("Panaxosides"[Title/Abstract] OR "Ginsenoside"[Title/Abstract])) OR ("Resveratrol"[MeSH Terms] OR ("3 5 4 trihydroxystilbene"[Title/Abstract] OR "3 4 5 trihydroxystilbene"[Title/Abstract] OR "trans-Resveratrol-3-O-sulfate"[Title/Abstract] OR "SRT-501"[Title/Abstract] OR "cis-Resveratrol"[Title/Abstract] OR "trans-Resveratrol"[Title/Abstract] OR "Resveratrol-3-sulfate"[Title/Abstract])) OR ("tanshinone"[Supplementary Concept] OR ("tanshinone i"[Title/Abstract] OR "TTE-50"[Title/Abstract] OR "tanshinone ii a"[Title/Abstract] OR "tanshinone iia"[Title/Abstract] OR "tanshinone ii b"[Title/Abstract] OR "tanshinone iib"[Title/Abstract])) OR ("tetramethylpyrazine"[Supplementary Concept] OR ("ligustrazine"[Title/Abstract] OR "TMPZ"[Title/Abstract] OR "chuanxiongzine"[Title/Abstract] OR "tetramethyl pyrazine"[Title/Abstract] OR "tetramethylpyrazine hydrochloride"[Title/Abstract] OR "Liqustrazine"[Title/Abstract])) OR "baicalin"[Supplementary Concept] OR "Berberine"[MeSH Terms] OR "rhodioloside"[Supplementary Concept] OR "total flavonoids of astragalus"[Title/Abstract] OR "Astragaloside"[Title/Abstract] OR "astragalus polysaccharide"[Title/Abstract] OR ("Emodin"[MeSH Terms] OR ("Rheum Emodin"[Title/Abstract] OR "emodin rheum"[Title/Abstract] OR "Frangulic Acid"[Title/Abstract] OR "Archin"[Title/Abstract] OR "Frangula Emodin"[Title/Abstract] OR "Casanthranol"[Title/Abstract] OR "Peristim"[Title/Abstract])) OR "panax notoginseng saponins"[Title/Abstract] OR ("Acid, Rosmarinic "[MeSH Terms] OR ("Rosmarinate "[Title/Abstract] OR "Rosemary Acid"[Title/Abstract] OR "Acid, Rosemary"[Title/Abstract] OR "Rosmarinic Acid, (R-(E))-Isomer"[Title/Abstract] OR "Rosmarinic Acid"[Title/Abstract] OR ("Paclitaxel"[MeSH Terms] OR ("Anzatax"[Title/Abstract] OR "NSC-125973"[Title/Abstract] OR "NSC-125973"[Title/Abstract] OR "NSC125973"[Title/Abstract] OR "Taxol"[Title/Abstract] OR "Taxol A"[Title/Abstract] OR "Bris Taxol"[Title/Abstract] OR "Taxol, Bris "[Title/Abstract] OR "Paclitaxel, (4 alpha)-isomer "[Title/Abstract] OR "Paxene "[Title/Abstract] OR "Praxel"[Title/Abstract] OR "7-epi-Taxol"[Title/Abstract] OR "7 epi Taxiol "[Title/Abstract] OR "Onxol "[Title/Abstract])  **Web of science**  TS=(‘Spinal Cord Injuries’ OR ‘Spinal cord injury’ OR ‘Spinal injury’ OR ‘Spinal Cord Trauma’ OR ‘cord trauma spinal’ OR ‘Spinal Cord Traumas’ OR ‘trauma spinal cord’ OR ‘myelopathy traumatic’ OR ‘Traumatic Myelopathies’ OR ‘Traumatic Myelopathy’ OR ‘injuries spinal cord’ OR ‘cord injuries spinal’ OR ‘cord injury spinal OR injury spinal cord’ OR ‘Spinal Cord Transection’ OR ‘Spinal Cord Laceration’ OR ‘laceration spinal cord’ OR ‘post traumatic myelopathy’ OR ‘post traumatic myelopathy’ OR ‘Post-Traumatic Myelopathies’ OR ‘contusion spinal cord’ OR ‘contusions spinal cord’ OR ‘cord contusions spinal’ OR ‘Spinal Cord Contusions’ OR ‘Spinal Cord Contusion’) AND TS=( Curcumin OR ‘turmeric yellow’ OR ‘yellow turmeric’ OR ‘curcumin phytosome’ OR Diferuloylmethane OR Ginsenosides OR Panaxosides OR Ginsenoside OR Resveratrol OR ‘3 5 4 trihydroxystilbene’ OR ‘3 4 5 trihydroxystilbene’ OR ‘trans-Resveratrol-3-O-sulfate’ OR SRT-501 OR ‘cis-Resveratrol’ OR ‘trans-Resveratrol’ OR ‘Resveratrol-3-sulfate’ OR tanshinone OR ‘tanshinone I’ OR ‘TTE-50’ OR ‘tanshinone ii a’ OR ‘tanshinone iia’ OR ‘tanshinone ii b’ OR ‘tanshinone iib’ OR tetramethylpyrazine OR ligustrazine OR TMPZ OR chuanxiongzine OR ‘tetramethyl pyrazine’ OR ‘tetramethylpyrazine hydrochloride’ OR Liqustrazine OR baicalin OR Berberine OR rhodioloside OR ‘total flavonoids of astragalus’ OR Astragaloside OR ‘astragalus polysaccharide’ OR ‘Emodin OR Rheum Emodin’ OR ‘emodin rheum’ OR ‘Frangulic Acid’ OR Archin OR ‘Frangula Emodin’ OR Casanthranol OR Peristim OR ‘panax notoginseng saponins’ OR Paclitaxel OR Anzatax OR ‘NSC-125973’ OR ‘NSC 125973’ OR NSC125973 OR Taxol OR ‘Taxol A’ OR ‘Bris Taxol’ OR ‘Taxol, Bris’ OR ‘Paclitaxel, (4 alpha)-Isomer’ OR Paxene OR Praxel OR ‘7-epi-Taxol’ OR ‘7 epi Taxol’ OR Onxol OR ‘Acid, Rosmarinic’ OR Rosmarinate OR ‘Rosemary Acid’ OR ‘Acid, Rosemary’ OR ‘Rosmarinic Acid, (R-(E))-Isomer’ OR ‘Rosmarinic Acid’) 644  **Embase**  #1: ‘Spinal Cord Injuries’ OR ‘Spinal cord injury’ OR ‘Spinal injury’ OR ‘Spinal Cord Trauma’ OR ‘cord trauma spinal’ OR ‘Spinal Cord Traumas’ OR ‘trauma spinal cord’ OR ‘myelopathy traumatic’ OR ‘Traumatic Myelopathies’ OR ‘Traumatic Myelopathy’ OR ‘injuries spinal cord’ OR ‘cord injuries spinal’ OR ‘cord injury spinal OR injury spinal cord’ OR ‘Spinal Cord Transection’ OR ‘Spinal Cord Laceration’ OR ‘laceration spinal cord’ OR ‘post traumatic myelopathy’ OR ‘post traumatic myelopathy’ OR ‘Post-Traumatic Myelopathies’ OR ‘contusion spinal cord’ OR ‘contusions spinal cord’ OR ‘cord contusions spinal’ OR ‘Spinal Cord Contusions’ OR ‘Spinal Cord Contusion’  #2. Curcumin OR ‘turmeric yellow’ OR ‘yellow turmeric’ OR ‘curcumin phytosome’ OR Diferuloylmethane OR Ginsenosides OR Panaxosides OR Ginsenoside OR Resveratrol OR ‘3 5 4 trihydroxystilbene’ OR ‘3 4 5 trihydroxystilbene’ OR ‘trans-Resveratrol-3-O-sulfate’ OR SRT-501 OR ‘cis-Resveratrol’ OR ‘trans-Resveratrol’ OR ‘Resveratrol-3-sulfate’ OR tanshinone OR ‘tanshinone I’ OR ‘TTE-50’ OR ‘tanshinone ii a’ OR ‘tanshinone iia’ OR ‘tanshinone ii b’ OR ‘tanshinone iib’ OR tetramethylpyrazine OR ligustrazine OR TMPZ OR chuanxiongzine OR ‘tetramethyl pyrazine’ OR ‘tetramethylpyrazine hydrochloride’ OR Liqustrazine OR baicalin OR Berberine OR rhodioloside OR ‘total flavonoids of astragalus’ OR Astragaloside OR ‘astragalus polysaccharide’ OR ‘Emodin OR Rheum Emodin’ OR ‘emodin rheum’ OR ‘Frangulic Acid’ OR Archin OR ‘Frangula Emodin’ OR Casanthranol OR Peristim OR ‘panax notoginseng saponins’ OR Paclitaxel OR Anzatax OR ‘NSC-125973’ OR ‘NSC 125973’ OR NSC125973 OR Taxol OR ‘Taxol A’ OR ‘Bris Taxol’ OR ‘Taxol, Bris’ OR ‘Paclitaxel, (4 alpha)-Isomer’ OR Paxene OR Praxel OR ‘7-epi-Taxol’ OR ‘7 epi Taxol’ OR Onxol OR ‘Acid, Rosmarinic’ OR Rosmarinate OR ‘Rosemary Acid’ OR ‘Acid, Rosemary’ OR ‘Rosmarinic Acid, (R-(E))-Isomer’ OR ‘Rosmarinic Acid’  #3. #1 AND #2 896  **CNKI/高级检索; CNKI / Advanced Search**  SU %= '脊髓损伤' AND (SU %= '姜黄素' OR SU %= '人参皂苷 ' OR SU %= '白藜芦醇' OR SU %= '丹参酮' OR SU %= '川芎嗪' OR SU %= '黄芩苷' OR SU %= '黄连素' OR SU %= '红景天苷' OR SU %= '黄芪总黄酮' OR SU %= '黄芪甲苷' OR SU %= '黄芪多糖' OR SU %= '大黄素' OR SU %= '三七皂苷' OR SU %= '紫杉醇OR SU %= '迷迭香酸') 392  **万方/高级检索; Wanfang database/ Advanced Search**  (脊髓损伤) and ((姜黄素) OR (人参皂苷) OR (白藜芦醇) OR (丹参酮) OR (川芎嗪) OR (黄芩苷) OR (黄连素) OR (红景天苷) OR (黄芪总黄酮) OR (黄芪多糖) OR (大黄素) OR (三七皂苷) OR (紫杉醇) OR (迷迭香酸)) 301  **CBM/高级检索; CBM / Advanced Search**  ‘脊髓损伤’[摘要:智能] AND (‘姜黄素’[摘要:智能] OR ‘人参皂苷’[摘要:智能] OR ‘白藜芦醇’[摘要:智能] OR ‘丹参酮’[摘要:智能] OR ‘川芎嗪’[摘要:智能] OR ‘黄芩苷’[摘要:智能] OR ‘黄连素’[摘要:智能] OR ‘红景天苷’[摘要:智能] OR ‘黄芪总黄酮’[摘要:智能] OR ‘黄芪多糖’[摘要:智能] OR ‘大黄素’[摘要:智能] OR ‘三七皂苷’[摘要:智能] OR ‘紫杉醇’[摘要:智能] OR ‘迷迭香酸’[摘要:智能]) 143 |
| --- |
